# Nitroxide-Based Contrast Agents for MRI Cancer Diagnostics: Progress, Limitations, and Perspectives

**DOI:** 10.3390/molecules31060942

**Published:** 2026-03-11

**Authors:** Dmitry Mitin, Alexey Chubarov

**Affiliations:** 1 Institute of Chemical Biology and Fundamental Medicine Siberian Branch of the Russian Academy of Sciences, Novosibirsk 630090, Russia; d.mitin@g.nsu.ru; 2 Department of Physics, Free University of Berlin, 14195 Berlin, Germany

**Keywords:** nitroxide, spin probes, organic radical contrast agent (ORCA), electron paramagnetic resonance (EPR), magnetic resonance imaging (MRI), relaxivity, Overhauser enhanced magnetic resonance imaging (OMRI)

## Abstract

Magnetic resonance imaging (MRI) is one of the most powerful non-invasive methods for cancer diagnostics. To enhance image contrast and, therefore, diagnostic accuracy, contrast agents (CAs) are widely used in clinics. For decades, the clinical standard has been metal-based CAs, primarily gadolinium- and manganese-based chelates, or iron oxide nanoparticles. However, metal-based CAs possess sub-effects, toxicity, and associated adverse health effects, such as nephrogenic systemic fibrosis. As an alternative, metal-free organic radical CAs (ORCAs), based on nitroxides, have been developed. ORCAs are widely used as primary ^1^H-MRI agents and offer many advantages, including high biocompatibility, biodegradability, and easy functionalization. Attachment of nitroxides to natural or synthetic polymers enables the development of constructs with prolonged systemic circulation time and tumor-targeted delivery. Furthermore, MR-signal amplification can be achieved through physical hyperpolarization techniques, such as dynamic nuclear polarization (DNP) and Overhauser-enhanced MRI (OMRI), in which nitroxide radicals serve as hyperpolarizing agents, yielding signal enhancements. This review summarizes low-molecular-weight nitroxides, polymeric, and biomacromolecular platforms for ^1^H-MRI, focusing on physicochemical properties, preclinical evidence in tumor imaging, and current limitations. One section highlights the use of nitroxides as hyperpolarizing agents for tumor metabolism analysis or OMRI. The review addresses ongoing challenges and outlines future perspectives for the clinical translation of ORCAs in cancer diagnostics.

## 1. Introduction

Effective cancer treatment requires a comprehensive approach that includes diagnostics, targeted therapy, and dynamic monitoring of disease progression [1]. Magnetic resonance imaging (MRI) is one of the most common non-invasive medical techniques for obtaining high-quality three-dimensional body images in the clinical diagnosis of cancer [2,3,4,5,6,7,8,9]. MRI is an essential technique for tumor detection with excellent spatial resolution and unlimited penetration depth [10,11]. Despite its good sensitivity and detection capabilities, the diagnostic efficacy of native ^1^H-MRI is limited by the similar morphological features of malignant and benign lesions and the low efficiency of metastasis detection [12]. ^1^H-MRI contrast agents (CAs) are widely used to improve the difference between the target tissue and the background signal. In a special category, heteronuclear MRI, CA for ^19^F- and ^31^P-NMR in vivo or MRI, which are primarily used for studies in cells or animal models [4,13,14,15,16], are not standard clinical methods and are not included in this review. The CA for ^1^H-MRI is based on the effect of paramagnetic species on the spin relaxation of water protons in tissue, mainly Gd- and Mn-complexes or iron oxide nanoparticles (MNPs) [3,6,17,18,19,20,21,22,23]. The spin relaxation of protons is characterized by two parameters called longitudinal (*T*_1_) and transverse (*T*_2_) relaxation times. Depending on their effect on these parameters, CAs fall into two classes_:_ *T*_1_- and *T*_2_-shortening agents, which produce positive and negative contrast in *T*_1_- and *T*_2_-weighted images, respectively [3,19,24,25]. Relaxivities *r*_1_ and *r*_2_ show the ability of CAs to change the relaxation rates 1/*T*_1_ and 1/*T*_2_ of water protons in dependence on the concentration. Relaxivities *r*_1_ and *r*_2_, expressed in units of mM^−1^s^−1^, clearly indicate the efficiency of CA in the registration of *T*_1_- and *T*_2_-weighted images [26,27].

Recent concerns about the toxicity of Gd-based CA and MNPs have limited and complicated their clinical applications [19,28,29,30,31,32,33]. Consequently, the Medicines Agencies of Europe and the USA recently recommended the use of gadolinium-based CA at the lowest possible dose [29,30,31]. Furthermore, unstable MNPs induced oxidative stress, reactive oxygen species generation, DNA damage, and, in some cases, a mutagenic effect [17,34,35,36,37]. Therefore, there is an impetus to develop alternatives to metal-containing CA. Organic radicals have recently been considered as possible CA for ^1^H-MRI [25,38,39,40,41]. Organic radical CA (ORCA) is used for primary ^1^H-MRI with standard protocols and has shown low systemic toxicity, biocompatibility, and biodegradability [25,38,41,42,43,44,45,46]. However, several drawbacks, such as rapid nitroxide reduction in vivo to diamagnetic species [47,48,49,50] and low relaxivity per radical (~0.15 mM^−1^s^−1^ at 7 T) [51,52], render them unsuitable for MRI. In recent decades, the joint efforts of scientists from various fields have enabled the solution of these and many other problems related to the use of nitroxides and have brought ORCA to a new level [13,41,53,54,55,56,57,58]. In this review, we focus on recent progress in the development of ORCAs, covering systems ranging from low-molecular-weight compounds to large multifunctional constructions based on synthetic polymers and biopolymers. These materials are considered promising metal-free alternatives to conventional Gd chelates and MNPs currently used in MRI. The review begins with a brief introduction to the general structure, classification, and physicochemical properties of nitroxides, as well as strategies to improve their stability and solubility. We discuss low-molecular-weight nitroxides and their chemical modification with targeting groups, such as amino acids and sugars, which can improve their biodistribution. Strong attention is also given to nitroxides conjugated to lipids that can self-assemble into nanocarriers, thereby improving nitroxide stability within the carrier and potentially enabling accumulation in tumor tissues. Subsequently, a strategy for increasing biostability, relaxivity, and blood circulation time of ORCAs by attaching nitroxides to linear, brush-arm star, and dendrimeric artificial polymers is provided. The key challenges associated with the design, synthesis, properties, and mechanism of action of ORCAs, including their stability, potential toxicity, and selective accumulation in tumor tissues, are presented, along with suggestions for improvement. Relaxivity parameters and selected MRI applications are summarized in tables to facilitate comparison between different systems. In addition, the perspectives of ORCAs for both *T*_1_- and *T*_2_-weighted MRI images are extensively discussed. A specific section highlights the use of nitroxides as hyperpolarizing agents for Overhauser-enhanced magnetic resonance imaging (OMRI), a technique capable of significantly increasing MRI sensitivity. We also focused on activatable OMRI probes that respond to enzymatic activity in cancer tissues or enable the investigation of tumor metabolism. Finally, we outline limitations and future directions for the development of ORCAs, highlighting key directions to improve their performance in cancer diagnostics.

## 2. Classification and Properties of ORCA

In this section, ORCA is divided into three types: (1) functionalized small molecular weight compounds, including self-assembled forming large species; (2) various artificial polymer-based systems, including linear, brush-arm star, and dendrimeric; and (3) biomacromolecule-based. For each class, representative CAs are discussed, along with key advances in improving relaxivity and resistance to reduction in vivo, as well as prospects for further optimization and potential clinical translation for cancer diagnostics.

### 2.1. Low-Molecular-Weight and Self-Assembled ORCA

**Figure 1:**
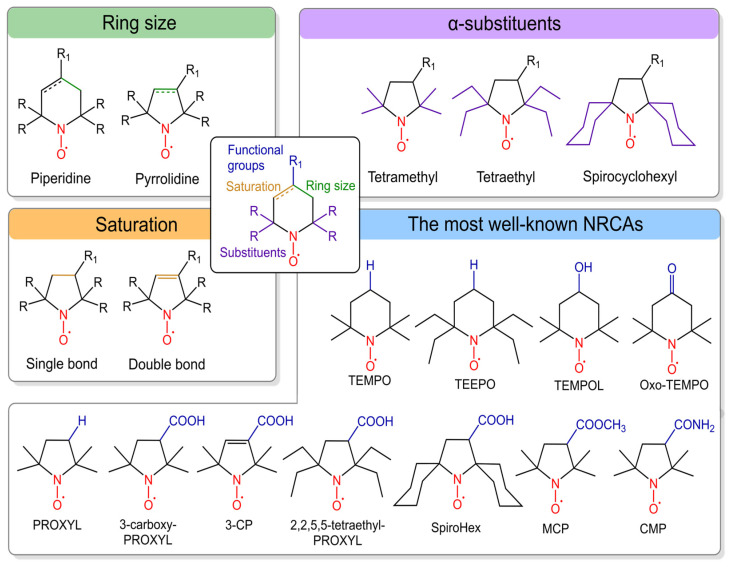
Structures of nitroxides for ORCA production.

Low-molecular-weight nitroxides face several key limitations in cancer studies, including rapid in vivo reduction, short blood circulation time, rapid elimination from the body, low relaxivity, and inability to accumulate in tumors [59]. Nevertheless, some well-soluble nitroxides, such as 2,2,6,6-tetramethylpiperidin-1-oxyl (TEMPO) and 2,2,5,5-tetramethylpyrrolidin-1-oxyl (PROXYL) derivatives (Figure 1), can be used as redox-sensitive probes that report local tissue redox status due to their ability to penetrate cell membranes, which is vital for the diagnosis of oncological diseases [60,61]. However, the analysis by *T*_1_-weighted MRI should be performed quickly within 5–15 min, because of the reduction of the radical [51,60]. This procedure is complicated, and the signal is very low. In this way, it cannot be considered a standard method in medical practice but rather something possible but difficult and in need of improvement. To avoid the problem of radical instability in vivo, a large series of nitroxides was synthesized, and their MRI performance was evaluated. Structures and properties of nitroxide-based ORCA used for MRI studies are presented in Figure 1 and Table 1.

Among all nitroxides, derivatives of the five-membered PROXYL [51,71,72] and the six-membered TEMPO [52,63] have received the widest application in preclinical studies. Their enhanced stability justifies the choice of cyclic nitroxide. The unpaired electron localized on the N–O. group is stabilized through overlap of the nitrogen and oxygen 2p^z^-orbitals, resulting in a high delocalization energy (23–30 kcal/mol) [73]. This delocalization strengthens the N–O^•^ bond compared to acyclic analogs, thereby increasing the overall radical stability. Additional protection against in vivo reduction by biological antioxidants is provided by steric shielding of the N–O^•^ group with bulky alkyl substituents at the α-positions relative to the nitrogen atom [74,75,76,77,78]. For example, a sterically shielded nitroxide has been developed, in which the methyl groups at the 2,6-positions of TEMPO or at the 2,5-positions of PROXYL are replaced with bulkier substituents, such as ethyl (Figure 1) [78]. For instance, in the presence of a 20-fold excess of ascorbate, the reduction rate exhibited by 2,2,5,5-tetraethyl-PROXYL is over 60 times slower than that of 3-carbamoyl-PROXYL. Similarly, the spirocyclohexyl-substituted PROXYL derivative (SpiroHex) retains up to 50% of its EPR signal after 3 h of incubation under the same reducing conditions. In contrast, conventional PROXYL is fully reduced within minutes [74]. The reduction reaction rate is associated with steric and energy stabilization of a nitroxide and its corresponding hydroxylamine. Synthesis of more sterically shielded pyrrolidine nitroxides with both ethyl and tert-butyl groups at each of the α-carbon atoms of the nitroxide moiety results in unexpectedly fast reduction due to the destabilization of the planar nitroxide moiety [75]. This data demonstrates the complexity of nitroxide design and should be taken into account. Although steric hindrance is a common strategy to reduce bioreduction, its efficiency depends heavily on conditions. It should be tested in model primary reductant systems, such as ascorbate or ascorbate/glutathione. However, these two systems show markedly different results for nitroxides with different steric hindrance. Tetramethyl-substituted nitroxides are rapidly reduced in both systems. Tetraethyl analogs show insignificant reduction with ascorbate but measurable reduction in the ascorbate/glutathione mixture [78]. Furthermore, because of the low solubility of some nitroxides, their reduction kinetics are studied in a water/organic solvent mixture. The kinetic interpretation is different, complicating the calculation of reduction constants and their comparison. All this leads to complete chaos in the literature, misrepresentation, and significant difficulties in the field’s progress. Unification of conditions and systems, along with comprehensive analysis, is required to take the area to the next level.

A significant challenge in designing sterically shielded nitroxides is their low solubility. For example, 2,2,6,6-tetraethylpiperidin-1-oxyl (TEEPO, Figure 1) exhibits good solubility in polar aprotic organic solvents (e.g., chloroform or DMSO) but is practically insoluble in water [79]. To cancel this restriction, chemical modification with hydrophilic groups that form H-bonds or ionic interactions, such as OH, NH_2_, and COOH, is required. However, this can only solve the problem to a restricted extent, limiting its application in biological systems. The aqueous solubility of nitroxides can be enhanced through chemical modification with hydrophilic biomolecules, such as amino acids (methionine, L-cysteine [79,80]) and carbohydrates (glucose, aminomannose [58,81]) (Table 2). For instance, conjugation of TEEPO with glucose (TEEPO-Glc) or methionine (TEEPO-Met) renders them soluble in a 10% (*v*/*v*) DMSO/water mixture, making them suitable for subsequent in vivo imaging experiments [79,82].

Relaxivity (*r*_1_ and *r*_2_) is the parameter that describes how strongly a substance changes the relaxation times *T*_1_ or *T*_2_ of water protons in tissues per CA concentration. The magnetic field strength is an essential parameter for relaxivity measurement. This dependence arises from the relationship between field B_0_ and the Larmor frequency (ω_0_), defined by ω_0_ = γB_0_, where γ is the gyromagnetic ratio [83,84]. The efficiency of paramagnetic relaxation is defined by the spectral density of magnetic field fluctuations J(ω), which depends on the ratio of the Larmor frequency to the correlation times of molecular motion (τ). Since J(ω) is frequency-dependent, any change in B_0_ alters the relaxivity [26,85]. The relaxivity changes with magnetic field strength are different for nitroxides, transition metals (e.g., Gd), MNPs, and small and large molecules. That is why experimental measurement of relaxivity is required for each CA in valuable field conditions. Unlike Gd chelates, where inner-sphere relaxation dominates, low-molecular-weight nitroxides in aqueous solution primarily relax water protons via an outer-sphere mechanism (~75% contribution), governed by the translational diffusion of water molecules around the paramagnetic center. This distinction in relaxation mechanism, and only one unpaired electron (S = 1/2), results in lower relaxivity values compared to Gd-based CAs, such as Gd-DTPA and Gd-DOTA (Table 1) [51,52]. A fundamental limitation of the inherently low relaxivity per nitroxide remains. The relaxivity of typical nitroxides ranges from 0.14 to 0.27 mM^−1^s^−1^ at magnetic fields of 1.5–11.7 T (Table 1). Nitroxides’ *r*_1_ is approximately an order of magnitude lower than that of clinically used Gd^3+^ chelates, which show *r*_1_ values of 3.5–4.5 mM^−1^s^−1^ at 1.5 T [19]. For small-molecular-weight nitroxides like TEMPO, the *r*_1_ value decreases rapidly with the field increase (e.g., see TEMPOL or MCP Table 1). Specifically, *r*_1_ values decrease from 0.35 to 0.45 mM^−1^s^−1^ at ultralow fields < 0.02 T to ~0.23 mM^−1^s^−1^ at 1.5 T, and further to ~0.12–0.14 mM^−1^s^−1^ at a high field > 7.0 T [63,86]. On the contrary, the *r*_2_ increases moderately, and *r*_2_/*r*_1_ increases sharply and becomes high only at a high field > 10 T. Thus, small nitroxides are weak *T*_1_ agents at a low field and mostly *T*_2_ agents at a high field, but much weaker than the primary used MNPs.

The same situation with low relaxivity was observed for nitroxides conjugated with biomolecules (Table 2). Conjugation of nitroxides with amino acids or carbohydrates should enable selective accumulation in tumor tissue, as cancer cells exhibit heightened uptake of these nutrients due to the Warburg effect and enhanced biosynthetic demands [79,81]. This process increases their local concentration in the tumor, enabling their detection by MRI. However, in vivo MRI studies have failed to confirm tumor-selective accumulation for either TEEPO-Glc [81]. In a murine C6 glioma model, both ORCA exhibited pharmacokinetics similar to those of Gd^3+^ chelates, including rapid systemic distribution, limited tumor specificity, and predominant accumulation in the liver and spleen [81].

**Table 2:** Functionalized nitroxide structure, relaxivity *r*_1_, and MRI applications.

Nitroxide	Magnetic Field Strength, T	*r*_1_, mM^−1^s^−1^	^1^H-MRI Application	Ref.
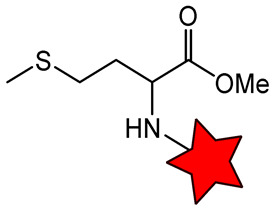 * TEEPO-Met	11.7	0.17	in vitro MRI phantoms	[79]
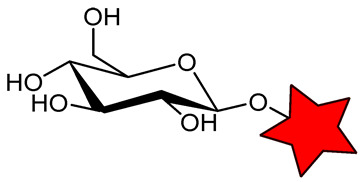 TEEPO-Glc	1.5	0.23	in vivo MRI of Wistar rats bearing C6 tumor	[81]
	9.4	0.13		
	11.7	0.12		
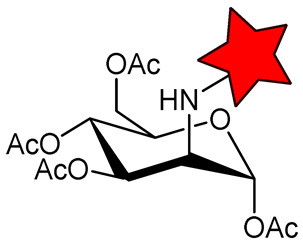 O-acetyl D-mannosamine-SpiroHex	-	-	in vivo MRI of C57BL/6 J mice	[58]
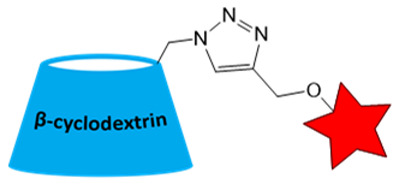 CD3	11.7	0.32	-	[87]
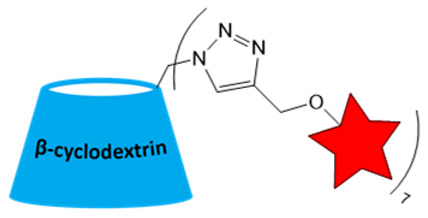 CD6	11.7	1.60	-	[87]
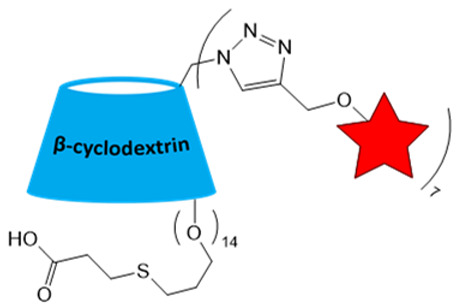 CD78	9.4	0.88	in vivo MRI of Wistar rats bearing glioma	[88]

* The star is a schematic representation of a nitroxide residue.

An additional strategy to increase the local concentration of nitroxides is to conjugate multiple radicals to a single nanoplatform. β-cyclodextrin (CD) was used to attach seven nitroxides, providing good solubility and *r*_1_ of 1.60 mM^−1^s^−1^ (Table 2). However, *r*_1_ was only 5 times higher than for CD with one nitroxide (0.32 mM^−1^s^−1^) [87]. Nevertheless, the increased number of hydrophobic nitroxides on the CD6 compromises its aqueous solubility. In subsequent studies [88], a strategy for functionalizing CD with thioglycolic acid was proposed, significantly improving its hydrophilicity. CD78 showed a decrease in *T*_1_ in vivo in the tumor, higher than TEMPOL but lower than Gd-DTPA (Table 1 and Table 2), enabling its use for in vivo ^1^H-MRI with further structure improvement [88].

An alternative strategy for developing ORCA involves using small-molecule compounds that self-assemble in aqueous solutions, forming different nanospecies (Table 3). Such systems include lipid and ureabenzoyl derivative (UBD). Self-assembly into nanospecies offers a key advantage: partial protection of the nitroxides from bioreduction. For example, RNP 2 showed significantly slower MR signal decay in vivo than TEMPOL, suggesting radical protection [89]. Nevertheless, these self-assembled systems still exhibit inherently low relaxivity (Table 3). Even the biradical system 2-HEG achieved only a ~2-fold increase in *r*_1_ relative to monomeric analogs [90]. Furthermore, thermal instability remains a critical limitation for lipid-based ORCA. At physiological temperature, 2-HEG-based nanoparticles tend to aggregate into micrometer-scale size [90]. Such large aggregates cannot effectively exploit the Enhanced Permeability and Retention (EPR) effect for tumor targeting and are instead rapidly cleared by renal filtration, substantially decreasing their diagnostic utility. Their interactions with blood components also need to be studied. In particular, human serum albumin (HSA) can bind many fatty residues, which can lead to the destruction of such systems [91,92].

While functionalization with biomolecules or self-assembling compounds addresses solubility and stability issues, it does not resolve the core limitations of low relaxivity and lack of tumor-selective accumulation. Promising future directions include combining multi-nitroxide loading with active targeting groups (e.g., sugars, folic acid (FA), peptides, etc.) and engineering nanocarrier architectures to prevent aggregation and enhance tumor delivery.

### 2.2. Polymer-Based ORCA

A highly effective strategy to enhance the clinical potential of ORCAs involves the covalent conjugation of nitroxides to synthetic polymers. The attachment of nitroxides to polymers increases relaxivity per molecule and prolongs blood circulation time, thereby improving MRI properties and enabling the transition to clinical MRI [41,94,95]. The increase in molecular weight and the encapsulation effect of polymer chains change the correlation time of the radical, which can increase *r*_1_ and *r*_2_ [95]. However, these changes in relaxivity and radical protection from reduction are highly dependent on the polymer’s three-dimensional structure. This subsection highlights various macromolecular platforms, including linear, brush-arm star, and dendrimeric polymers, including their properties, such as enhanced relaxivity, improved biostability, and efficacy to serve as CAs for MRI in vivo [96,97,98].

#### 2.2.1. Linear Polymer-Based ORCA

**Figure 2:**
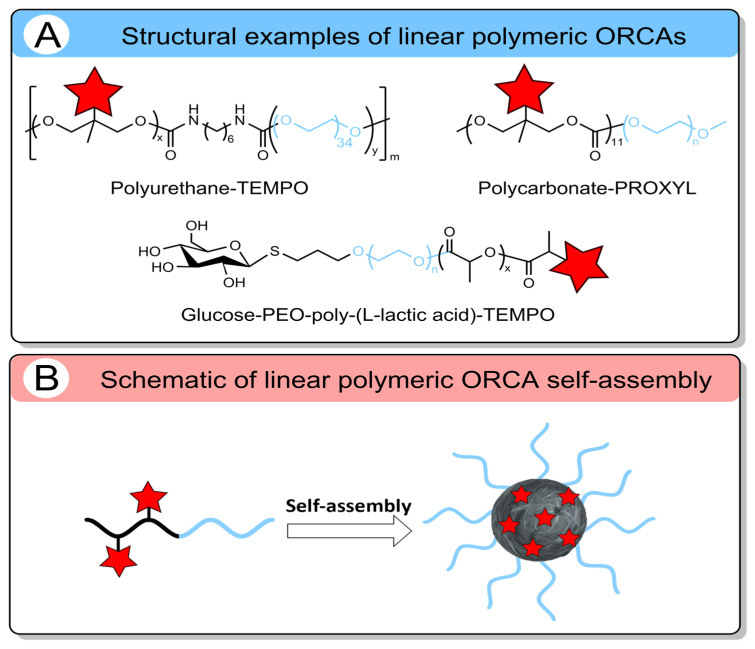
(**A**) Structures of linear polymeric ORCAs. (**B**) Schematic representation of linear polymeric ORCAs self-assembly. The red star represents a nitroxide residue. Blur color of the chain means PEG.

Linear polymerization represents one of the simplest methods for constructing polymeric carriers, with demonstrated utility in both therapeutic and diagnostic delivery systems [99,100]. Linear polymeric ORCAs are classified mostly according to the type of polymer backbone, such as polyurethanes [101], polycarbonates [96], and poly(L-lactic acid) [102] (Figure 2A).

The second type of classification is by nitroxide functionalization: (1) polymerization of monomers pre-functionalized with nitroxyl radicals [101]; (2) post-polymerization modification [96,102]. The first approach has been successfully employed to synthesize linear polyurethane-based ORCAs [101]. The second strategy is widely adopted due to its much easier synthesis of polymerization precursors. Further functionalization with an active nitroxide derivative is relatively straightforward and poses no particular difficulties [96,102]. For instance, pentafluorophenyl ester activation has been used to functionalize polycarbonate [96], while carbodiimide-mediated binding has enabled the conjugation of nitroxides to poly(L-lactic acid)-based polymers [102].

Linear polyurethane (PU)-based ORCA could be synthesized with a high TEMPO content [95]. The *r*_1_ and *r*_2_ reached 0.66 and 0.98 mM^−1^s^−1^ at 1.5 T, respectively (Table 4). Unfortunately, the PU-5 formulation exhibited poor solubility and self-assembly, yielding ~70 nm particles in aqueous solution (Figure 2B). In contrast, higher-MW PU-6 and PU-7 were highly monodisperse, with a size of 100 nm, but lower relaxivities *r*_1_ of 0.45 and 0.16 mM^−1^s^−1^ at 1.5 T, respectively. The same aggregation situation was obtained for amphiphilic poly(ethylene glycol)-b-polycarbonate-based diblock copolymers [96]. This design enables self-assembly into micellar nanostructures in aqueous solution with a size of 103 nm (Figure 2B). The nitroxide radicals are sequestered within the hydrophobic core, while the PEG chains form a hydrated outer shell [96]. That is why the obtained *r*_1_ value reached only 0.22 mM^−1^s^−1^ at 3 T (Table 4). To address insufficient MRI contrast and enhance tumor accumulation, glucose is used as a targeting ligand for poly(L-lactic acid)-based micelles (PPT@IC). MRI in mice 12 h after injection showed greater contrast enhancement in the tumor with glucose-functionalized GPPT@IC (65% signal increase) than with unmodified PPT@IC (15% signal increase) [102]. These studies demonstrate that surface modification with tumor-specific ligands enables greater tumor accumulation and a significant increase in signal intensity. Polymer structure can incorporate additional imaging capabilities, such as the incorporation of a fluorescent dye. This combination allows preoperative tumor localization via MRI, followed by intraoperative fluorescence-guided surgery. In preclinical studies, fluorescence imaging enables highly sensitive longitudinal tracking of tumor accumulation, biodistribution, and clearance in animal models [96,103,104].

ORCAs for MRI are not limited to conventional micelle-forming linear polymers. They can also be designed as three-dimensional hydrogel scaffolds based on linear polymer networks [105] (Table 4). Fabrication of hydrogels by crosslinking nitroxide-functionalized PEG, branched poly(acrylic acid), and agarose via ester bond formation, and by inserting TEMPO into the hydrogel matrix, leads to *r*_1_ of ~0.3 mM^−1^s^−1^ at 7.0 T. The hydrogel was tested in an in vivo mouse model by injection onto the spinal cord. The MRI after 1 and 6 months still showed a paramagnetic signal [105]. Owing to their high biocompatibility, prolonged half-life, and localized retention, hydrogel-based ORCAs hold significant promise for localized MRI diagnostics.

A key limitation of polymeric ORCAs is their pronounced hydrophobicity, primarily due to the high density of nitroxide moieties, which can decrease aqueous solubility and promote aggregation. Excessive incorporation of hydrophobic nitroxides can compromise aqueous solubility, thereby limiting self-assembly, which is essential for in vivo stability and efficiency. These results highlight that the rational design of high-relaxivity polymer-based ORCAs requires a balance between maximizing nitroxide loading and maintaining sufficient hydrophilicity. Consequently, while linear polymer-based ORCAs typically achieve only a ~2-fold increase in *r*_1_ compared to low-molecular-weight ORCAs (Table 4), their effectiveness remains significantly inferior to that of clinically used gadolinium-based chelates. Further optimization of polymer architecture, nitroxide amount, and surface hydrophilicity is required to address the gap in relaxometric performance.

#### 2.2.2. Brush-Arm Star Polymer-Based ORCA

**Figure 3:**
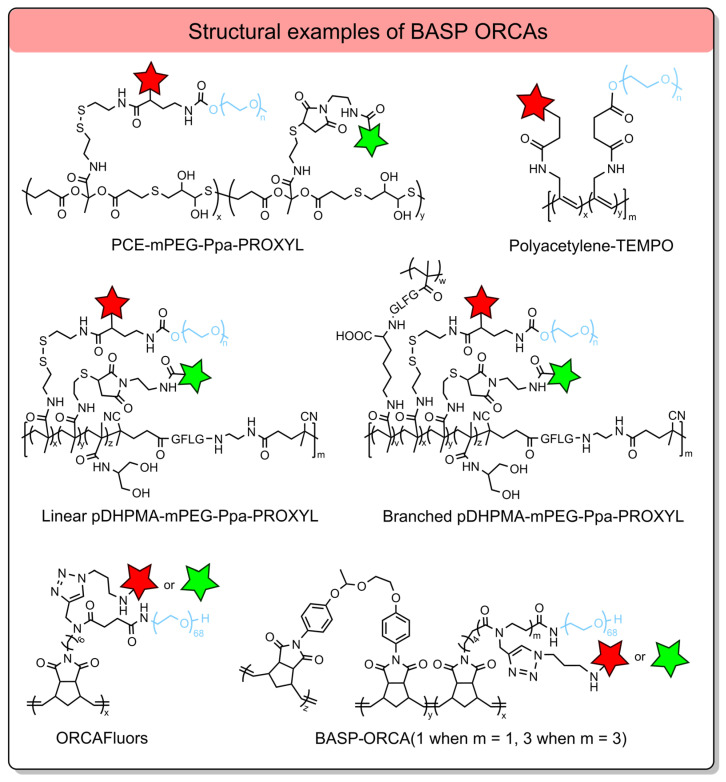
Structures of BASP ORCAs. The red and green stars are a nitroxide residue and fluorescent dye, respectively. Blur color of the chain means PEG.

Brush-arm star polymers (BASPs) are typically synthesized via polymerization of pre-functionalized monomers, which integrate two or more distinct functional components into a single polymer structure (Figure 3) [96,98].

Primary, these polymers integrate multiple functional components into a single nanostructure: a structural backbone, a nitroxide for MRI contrast, hydrophilic poly(ethylene glycol) (PEG) segments, and, optionally, fluorescent dyes (e.g., Cy5.5) for fluorescence-guided cell experiments or fluorescence imaging [106]. Common polymeric backbones used in BASP synthesis include polyacetylenes [107], polycarboxylates [108], polymethacrylamides [94,109], and polynorbornenes [38,106,110] (Figure 3). As with all high-MW polymers, BASPs exhibit prolonged blood circulation times. The BASP structure has a significantly higher loading of nitroxide radicals per molecule than the linear polymer, thereby increasing the construction’s relaxivity. In aqueous environments, BASPs self-assemble into micellar nanostructures, in which the hydrophobic domains with nitroxides are sequestered in the core, while PEG forms a hydrated layer. This process enhances solubility, reduces nonspecific interactions with blood components, and protects nitroxide radicals from bioreduction [107]. Among reported systems, the highest *r*_1_ per radical, 4–5 times that of the PROXYL radical, was achieved with polycarboxylate (PCE)- and linear polymethacrylamide (pDHPMA)-based ORCAs (Table 5). This enhancement arises from the slowing of nitroxide correlation time due to the high MW of the polymers, and sufficient chain flexibility ensures efficient water exchange around the paramagnetic centers. Interestingly, for branched pDHPMA-mPEG-Ppa-PROXYL, the *r*_1_ per radical decreased to 0.50 mM^−1^s^−1^ compared to the same linear polymer [102]. It can be explained by excessive chain rigidity and dense crosslinking, which restricts segmental mobility and hinders water diffusion to encapsulated nitroxyl radicals. Norbornene-based bottle brush polymers (ORCAFluors) displayed a lower *r*_1_ of 0.32 mM^−1^s^−1^ per radical but a significantly higher *r*_2_ of 0.82 mM^−1^s^−1^, yielding an *r*_2_/*r*_1_ ratio of 2.6 (Table 5) [106]. The much higher radical loading led to a total *r*_1_ and *r*_2_ per particle of 37 and 95 mM^−1^s^−1^, respectively (Table 5). These values are much greater than for *T*_1_ CA Magnevist (*r*_1_ = 3.1 mM^−1^ s^−1^ at 7 T) and *T*_2_ CA based on iron oxide Feraheme nanoparticles (*r*_2_ = 68 mM^−1^ s^−1^ at 7 T) (Table 1 and Table 5).

The problem of low relaxivity was finally solved by synthesizing the BASP ORCA family (Table 5). BASP production was optimized by incorporating a crosslinking agent during polymerization. The resulting BASP-ORCA1 with 92 nitroxide radicals per polymer exhibited enhanced high per-radical relaxivity due to improved steric shielding of the nitroxides within the densely packed brush structure [38]. For BASP-ORCA1, *r*_1_ and *r*_2_ were found as 37.6 mM^−1^s^−1^ and 428.8 mM^−1^s^−1^, respectively. Further increasing the nitroxide loading to over 200 radicals in BASP-ORCA3 led to a 1.5-fold rise in *r*_1_ per radical, translating to a BASP *r*_1_ of 126 mM^−1^s^−1^ per particle and an extraordinary *r*_2_ > 1000 mM^−1^s^−1^. It should be noted that, for a branched structure, crosslinking had a strong effect on transverse relaxivity, and *r*_2_ becomes higher than *r*_1_. Such changes mean increased potential for recording *T*_2_-weighted images. For well-known *T*_2_-CAs, Feraheme, and Resovist, *r*_2_/*r*_1_ ratios at 3 T are 8.8 and 19.5, respectively. The *r*_2_/*r*_1_ ratios for BASP-ORCA1 and BASP-ORCA3 are 11.4 and 7.3, respectively, indicating a high potential for these polymers in *T*_2_-but not *T*_1_-weighted images [38,41]. The *r*_2_ value of BASP-ORCA3 is an order of magnitude higher than that of commonly used iron oxide nanoparticles for *T*_2_-weighted MRI (cf. Table 5) [115].

**Figure 4:**
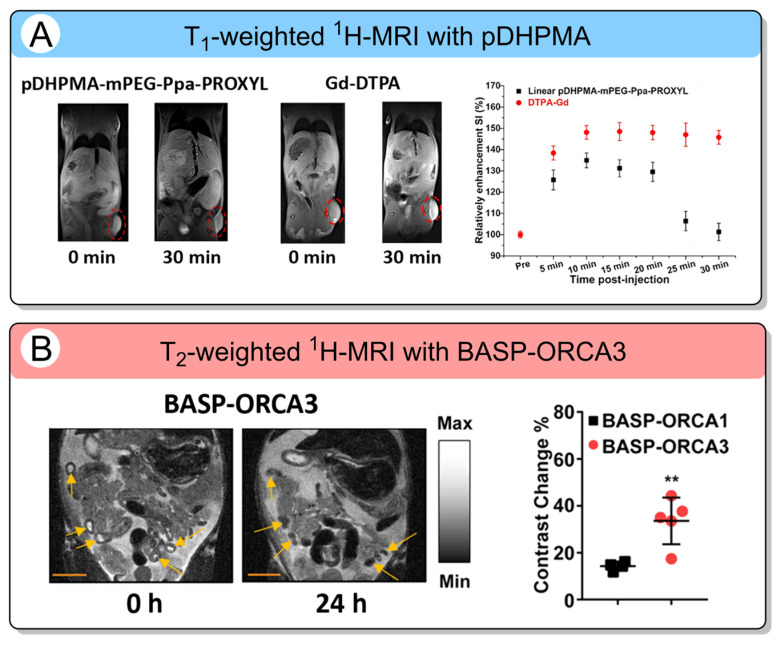
(**A**) Left: T_1_-weighted ^1^H-MRI of pDHPMA-mPEG-Ppa-PROXYL; Right: Enhanced signal ratio at the tumor after injection of pDHPMA-mPEG-Ppa-PROXYL or Gd-DTPA [96]. The red dashed circles indicate the tumor region. Copyright 2021, BioMed Central. (**B**) Left: T_2_-weighted ^1^H-MRI with BASP-ORCA3; Right: Enhanced signal ratio at the tumor generated by BASP-ORCA1 vs. BASP-ORCA3 [41]. Yellow arrows indicate tumors peritoneal organs. Statistical comparisons were performed with the Mann−Whitney U-test (** *p* ≤ 0.05). Copyright 2018, American Chemical Society (Washington, DC, USA).

These properties establish BASP-ORCA3 as a highly promising CA for sensitive tumor detection. Results of in vivo MRI studies have been conducted for linear polymer pDHPMA-mPEG-Ppa-PROXYL [94] (Figure 4A) and BASP-ORCA3 [41] (Figure 4B). After 20 min of injection, pDHPMA-mPEG-Ppa-PROXYL produced 34% *T*_1_-weighted signal enhancement in the tumors, whereas Gd-DTPA increased the signal by 50%. After this, the polymer slowly decreased, while the Gd-DTPA signal remained nearly unchanged for a longer period. For BASP-ORCA1 and BASP-ORCA3, tumor enhancement signals of 12.4% and 24.3%, respectively, were observed after 24 h.

These results indicate that increasing the loading of nitroxides per polymer particle enhances *T*_2_ contrast efficacy. However, for a stronger contrast effect, targeting of polymers to the tumor is required. For active receptor-mediated uptake, polymer surface functionalization with tumor-targeting ligands is useful. For example, the conjugate of polyacetylene-TEMPO with folic acid for targeting folate-receptor-overexpressing HeLa cells in BALB/c mice with subcutaneous tumors showed 43% tumor signal enhancement, compared with 29% for the non-targeted polymer after 2 h post-injection [107].

Nitroxide-based BASP simultaneously enhances four parameters essential for in vivo MRI CAs: (1) stability against bioreduction via polymer chain shielding; (2) prolonged circulation and passive tumor targeting through optimized hydrodynamic size; (3) high solubility by introduction into structure PEG polymer; (4) significantly amplified relaxivity due to the slowed rotational correlation time of the nitroxides in the macromolecular assembly and the high amount of nitroxides per molecule. BASP ORCAs represent a promising platform for future development, in combination with structural optimization and tumor-selective targeting strategies tailored to specific cancer types.

#### 2.2.3. Dendrimeric Polymer-Based ORCA

**Figure 5:**
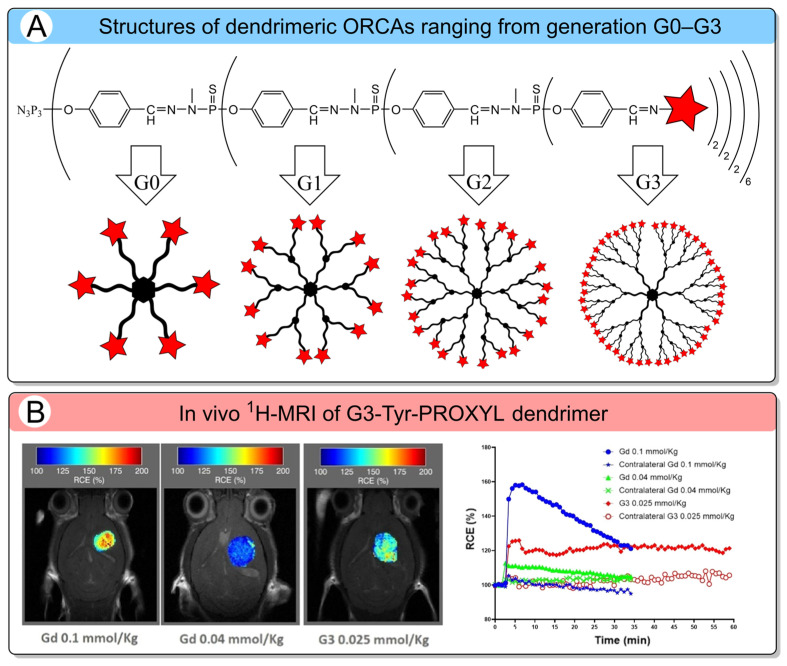
(**A**) Structures of G*n*-Tyr-PROXYL (*n* = 0–3) dendrimer. The red star represents a nitroxide residue. (**B**) Left: *T*_1_-weighted ^1^H-MRI with color-code scale (Gd 0.1 and 0.04 mmol/kg, 30 min; and G3 radical dendrimer 0.025 mmol/kg, 60 min). Right: kinetics of G3-Tyr-PROXYL accumulation in tumors [94]. Copyright 2022, American Chemical Society.

Dendrimers constitute a distinct class of polymers distinguished from conventional macromolecules by their controlled, hierarchical structure. Unlike linear polymers or BASP, dendrimers exhibit high monodispersity, predictable molecular sizes, and a dense surface arrangement of functional groups [97]. Their structure is assembled stepwise, generation by generation, enabling precise control over the number of terminal functionalities (Figure 5A). 

This synthesis strategy offers unique opportunities for chemical modification through reactive surface groups [66,95]. To date, a variety of ORCAs have been synthesized and evaluated using diverse dendrimer backbones, including polyamidoamines (PAMAM) [116], polypropylenimines (PPI) [44], polyphosphorhydrazones (PPH) [65,95], and poly(L-lysine) [117], as well as oligoethylene glycol [66] derivatives (Figure 5A). A major challenge in the application of higher-generation dendrimers as ORCAs is their poor aqueous solubility, which stems from the hydrophobic nature of the nitroxide radicals densely attached to their surfaces. PAMAM dendrimers ranging from generation G0–G4 were subsequently functionalized with nitronylnitroxide radicals [116]. The resulting dendrimer-based ORCAs exhibited a low per-radical relaxivity *r*_1_ of 0.11–0.13 mM^−1^s^−1^ at 0.5 T (Table 6), which can be attributed to aggregation in aqueous solutions due to limited water solubility. The poor water solubility is especially problematic for higher-generation dendrimers (G3–G4), which are of particular interest owing to their high nitroxide loading capacity and consequently elevated spin concentration. To overcome this critical issue for in vivo applications, several strategies have been developed that involve modifying high-generation dendrimers with hydrophilic moieties. One widely adopted approach involves conjugating water-soluble groups such as PEG chains to the dendrimer surface [44,117].

In the case of poly(L-lysine)-based dendrimers [117], two distinct PEGylation strategies were explored: (1) separate conjugation of nitroxide radicals and PEG chains to the dendrimer surface (PEG-PRx-KG6) and (2) use of PEG derivatives pre-functionalized with terminal nitroxide groups (PRx-PEG-KG6). The PEG-PRx-KG6 displayed a higher per-radical *r*_1_ 0.8 mM^−1^s^−1^ compared to PRx-PEG-KG6 0.5 mM^−1^s^−1^, due to enhanced steric shielding of nitroxides (Table 6). However, the total number of nitroxide radicals to be attached to the dendrimer surface is reduced, and the overall molecular relaxivity is lower than that achievable with a fully nitroxide-decorated dendrimer. To address this issue, subsequent studies developed fully water-soluble dendrimers based on polyphosphorhydrazones (PPH) and oligoethylene glycol (OEG), enabling complete functionalization with nitroxide radicals. Based on PPH scaffolds, a series of dendrimers designated G*n*-Tyr-PROXYL (*n* = 0–3) was synthesized, utilizing tyrosine as a bifunctional linker between dendrimer branches [65]. The amino group of tyrosine provided a site for nitroxide conjugation, while its carboxylic acid group—upon hydrolysis—imparted a negative charge, thereby ensuring high aqueous solubility. Dendrimers G*n*-OEG-PROXYL (*n* = 0, 1) were used, with diethylenetriaminepentaacetic acid as the core branching unit and OEG chains as linkers [66]. Its design enabled full-surface functionalization with PROXYL radicals, while maintaining water solubility. Notably, per-radical longitudinal relaxivities *r*_1_ for G3-Tyr-PROXYL (bearing 48 nitroxides) and G1-OEG-PROXYL (bearing 20 nitroxides) were measured at 0.27 and 0.17 mM^−1^s^−1^, respectively, at 7 T [65]. These values are comparable to those of low-molecular-weight nitroxides. The overall molecular *r*_1_ reaches 13 mM^−1^s^−1^, approximately four times higher than for Gd-DTPA chelate (Table 6). Moreover, *r*_2_*/r*_1_ values are about 1–1.2, indicating the *T*_1_-weighted image behavior of this dendrimer. The in vivo contrast-enhancing potential of the water-soluble dendrimer G3-Tyr-PROXYL was evaluated in GL261 glioblastoma-bearing mice (Figure 5B) [95]. Following a low-dose intravenous injection (0.025 mmol/kg), it provided tumor > 20% contrast enhancement without observable toxicity. Notably, G3-Tyr-PROXYL demonstrated selective accumulation in brain tumor tissue and prolonged retention ≥ 2.5 h. Moreover, the dendrimer-conjugated radicals exhibited remarkable stability in biological environments. Thus, these properties, such as high spin concentration and tumor selectivity, make fully functionalized, water-soluble dendrimers a highly promising alternative to metal-based CAs, especially for glioblastoma MRI.

### 2.3. Biomacromolecule-Based ORCA

**Figure 6:**
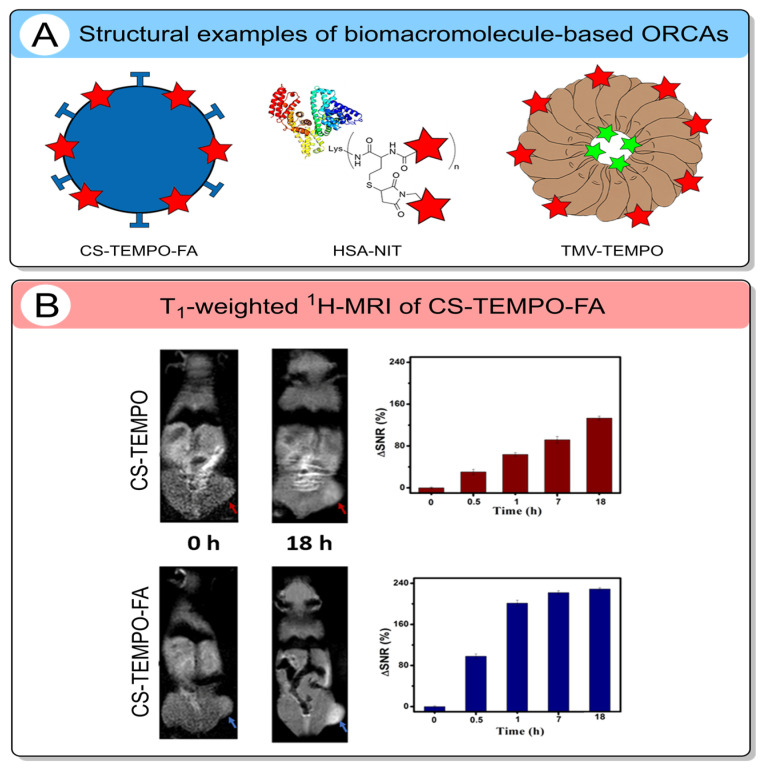
(**A**) Structures of biomacromolecule-based ORCA. A heart-like protein structure in the middle means albumin. The red stars are nitroxide residues. (**B**) Left: *T*_1_-weighted ^1^H-MRI with CS-TEMPO and CS-TEMPO-FA. Right: Enhanced signal ratio at the tumor generated by CS-TEMPO and CS-TEMPO-FA [119]. Red and blue arrows indicate tumor locations of CS-TEMPO and CS-TEMPO-FA in MRI, respectively. Copyright 2021, American Chemical Society. The red and green stars represent a nitroxide residue and fluorescent dye, respectively.

Biomacromolecules such as polysaccharides [46,119], proteins [42,120], and viral nanoparticles [121,122] represent a highly promising platform for developing ORCAs for MRI-based cancer diagnostics (Figure 6A).

In contrast to synthetic polymers, biopolymers exhibit exceptional biocompatibility, inherent biodegradability, and low immunogenicity, thereby minimizing toxicity risks and significantly prolonging circulation time [123,124]. Moreover, many biomacromolecules possess abundant and diverse functional groups that enable efficient conjugation of a high density of nitroxides, thereby substantially enhancing the relaxivity of the resulting ORCAs [120]. An additional advantage is their biological functions, such as the ability to cross the blood–brain barrier and to accumulate in tumors via the EPR effect or active targeting through receptor-mediated uptake [42]. These properties make biomacromolecular platforms suitable for the rational design of metal-free ORCAs.

In Section 2.1, low-molecular-weight ORCAs based on biomolecules, such as amino acids, glucose, and cyclodextrins, were discussed [81,88]. While these agents improved nitroxide solubility and exhibited good biocompatibility, they suffered from significant limitations, including low per-radical relaxivity (e.g., *r*_1_ TEEPO-glucose 0.23 mM^−1^s^−1^) and, in most cases, a lack of selective tumor accumulation. To address these disadvantages, a promising strategy is to develop biopolymer-based ORCAs that increase the number of nitroxide moieties per macromolecule and enhance tumor accumulation.

One approach uses glycol chitosan (GC), a widely employed and biocompatible polysaccharide derivative [125,126]. GC was used to synthesize tumor-targeted nanoparticles (CS-TEMPO-FA) with an optimal hydrodynamic diameter of 113 nm [46]. These nanoparticles were functionalized with TEMPO nitroxide and FA to enable receptor-mediated delivery to folate-receptor-overexpressing tumors. Notably, the compartmentalization of nitroxides within the hydrophilic GC shell enhanced their stability against bioreduction. Enhanced stability was quantitatively confirmed by kinetic analysis of ascorbate-mediated reduction, revealing more than a 128-fold decrease in reduction susceptibility for CS-TEMPO-FA compared to TEMPO. The CS-TEMPO-FA nanoparticle achieved a per-radical longitudinal relaxivity *r*_1_ of 1.58 mM^−1^s^−1^ at 1.5 T, 5-fold higher than that of conventional low-molecular-weight nitroxides (Table 7). Further optimization of the structure and incorporation of up to 161 TEMPO radicals per nanoparticle results in high molecular relaxivities of *r*_1_ = 254 mM^−1^s^−1^ and *r*_2_ = 340 mM^−1^s^−1^ [119]. The *r*_2_*/r*_1_ value is 1.3, indicating a *T*_1_ MRI image behavior. To validate its contrast-enhancing capability and tumor-targeting efficiency, CS-TEMPO-FA was employed for in vivo MRI of tumor-bearing mice (Figure 6B) [119]. Following intravenous injection, a significant increase in MR signal intensity was observed in the tumor region, with 229% for CS-TEMPO-FA and 134% for the non-targeted (no FA) control CS-TEMPO, persisting for up to 17 h. This pronounced and sustained enhancement demonstrates the effective active tumor targeting mediated by FA conjugation. These findings highlight the potential of CS-TEMPO-FA as a promising metal-free CAs.

One promising protein-based biomacromolecule scaffold is the rod-shaped capsid of tobacco mosaic virus (TMV). Composed of 2130 coat proteins, TMV forms a rigid cylindrical structure measuring 300 × 18 nm, with surface-exposed tyrosine and glutamic acid residues amenable to chemical modification. Nitroxides conjugated to TMV, yielding an ORCA with high per-radical relaxivities *r*_1_ 1.5 mM^−1^s^−1^ at 1.5 T, comparable to chitosan- and albumin-based ORCAs and *r*_2_ values 1.5–2-fold higher [121]. In another study, a supramolecular strategy to further enhance stability by forming a pseudorotaxane complex between TMV-TEMPO and cucurbit [8] uril was employed [122]. The resulting steric shielding by cucurbit [8] uril improved resistance to bioreduction and yielded an *r*_1_ of 1.9 mM^−1^s^−1^ at 1.0 T. This provided *T*_1_-weighted contrast enhancement in BALB/c mouse muscle tissue that persisted for up to 2 h post-injection.

**Figure 7:**
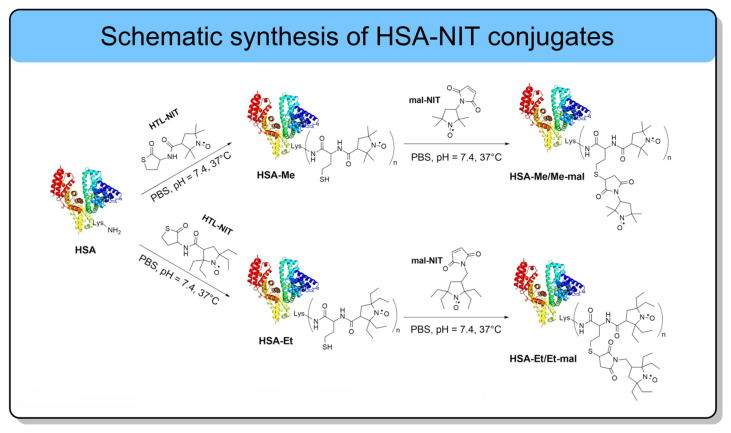
HSA-NIT conjugate synthesis. HSA is shown schematically as a color helical ribbon structure (reproduced from Int. J. Mol. Sci. 2024, 25, 4041 [120]); open access article distributed under the terms and conditions of the Creative Commons Attribution (CC BY 4.0).

Human serum albumin (HSA) has emerged as a highly promising biomacromolecule scaffold for the development of advanced diagnostic and theranostic agents [127,128,129]. HSA is the most abundant plasma protein and plays a central role in the transport of a wide range of endogenous and exogenous molecules [91,129]. Owing to its exceptional biocompatibility, long blood half-life, tumor-targeting capability by interaction with albumin-binding receptors, and its ability to cross biological barriers, HSA contains a single free cysteine residue (Cys34) and 59 lysine residues, both of which can be exploited for covalent conjugation with nitroxides. Importantly, HSA maintains its structural integrity despite chemical modification, particularly when site-selective strategies are employed [42]. HSA was site-selectively functionalized with nitroxide derivatives at lysine residues of 525, 212, 205, 159, 137, 12, and 4, as confirmed by MALDI-ToF mass spectroscopy [42]. It was achieved using a physiologically *N*-homocysteinylation reaction with homocysteine thiolactone derivatives of normal and sterically hindered nitroxides. Chemical modification was adapted to occur at the same lysine residues labeled in the in vivo reaction between albumin and homocysteine thiolactone. Importantly, the conjugate retained a native-like secondary structure of HSA, exhibiting only a minor reduction in α-helical content from 55% to 46–49% with the same amount of β-sheets and no oligomer formation by SDS-PAGE. The nitroxide radicals attached to albumin could be classified based on their rotational correlation time (τ_c_) into two populations: “fast” radicals with a τ_c_ of 2–3 ns and “slow” radicals with a τ_c_ of 10–14 ns. The authors hypothesize that the “fast” nitroxides are located on the protein surface in a solvent-exposed environment, whereas the predominant “slow” nitroxides reside in sterically hindered sites. For these conjugates, HSA–NIT, four types of nitroxides (two tetramethyl and two tetraethyl pyrrolidines) were used to evaluate the stability and relaxivity of the different radicals upon attachment to the protein. HSA–NIT exhibited per-radical relaxivities of *r*_1_ 0.33–0.51 mM^−1^s^−1^ and *r*_2_ 4.7–7.2 mM^−1^s^−1^ at 25 °C and 7.0 T, with corresponding per-molecule *r*_1_ 0.8–2.24 mM^−1^s^−1^ and *r*_2_ 11.2–27.9 mM^−1^s^−1^. The high *r*_2_*/r*_1_ ratio of 10.4–14.3 indicated strong transverse relaxation dominance, positioning these HSA–NIT conjugates as promising *T*_2_-weighted ORCAs for MRI [42]. It was found that attachment to the protein highly stabilized both tetramethyl and tetraethyl nitroxides. For example, the reduction constant for tetramethyl nitroxide changed from k = 0.22–0.58 M^−1^s^−1^ to 0.096 M^−1^s^−1^. For the tetraethyl radicals, the reduction constants were found to be k = 0.0018–0.0022 M^−1^s^−1,^ which is an order of magnitude slower than the nitroxides on a dendrimer-based ORCAs (k = 0.038 M^−1^s^−1^) [38]. Cell viability in breast cancer MCF-7 and human glioblastoma T98G cells shows no significant difference compared with native HSA. This finding underscores the need to further optimize the conjugate, particularly by increasing the number of nitroxide radicals per HSA molecule, to enhance its MRI diagnostic efficiency, as demonstrated in subsequent work (Table 7) [120]. To overcome the limitation of low radical loading, a new generation of HSA-NIT [120] developed an optimized dual-functionalization strategy that combined homocysteine thiolactone and maleimide derivatives of nitroxides (Figure 7). This approach enabled dual labeling of a single lysine residue via thiolactone-mediated acylation, followed by maleimide conjugation to the newly formed thiol group, thereby preventing disulfide-mediated oligomerization and increasing spin concentration to ~20 nitroxide residues per HSA molecule (Figure 7). Extensive protein modification typically induces significant conformational changes. However, in the case of albumin functionalized with homocysteine thiolactone and maleimide nitroxide derivatives at a loading of approximately 20 molecules per protein, only moderate structural alterations were observed. It should be noted that two nitroxides modify one lysine residue, which results in a less dramatic change in structure (Figure 7). These included a reduction in α-helical content to ~43–44%, accompanied by a slight 3–4% increase in β-sheet content. Importantly, this degree of modification did not induce protein oligomerization by SDS-PAGE or cytotoxicity, confirming the biocompatibility and structural integrity of the highly loaded HSA–NIT conjugates. The study employed both methyl- (Me) and more sterically hindered ethyl- (Et) substituted nitroxide derivatives to evaluate the impact of substituent bulk on stability.

**Figure 8:**
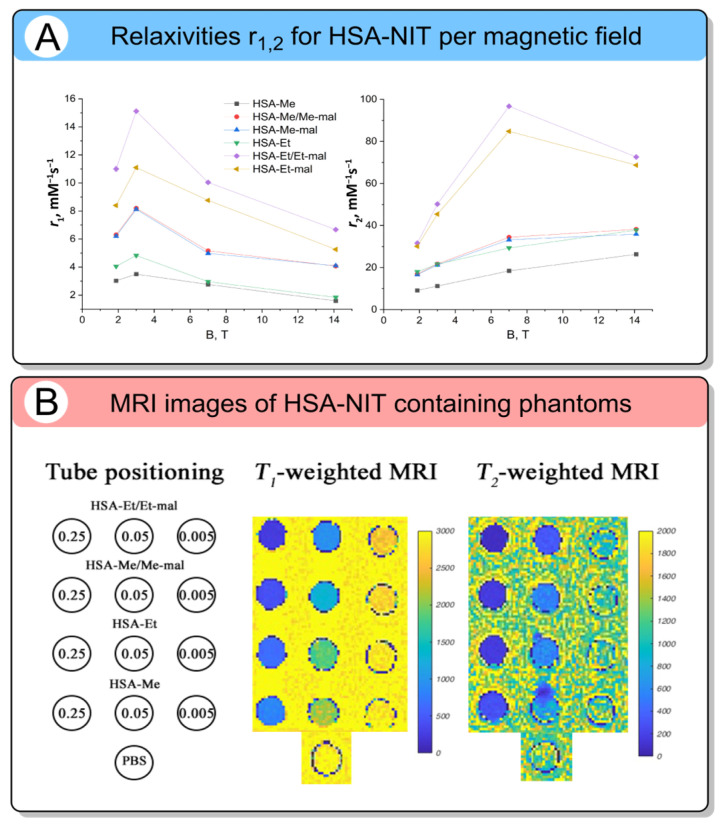
(**A**) Relaxivities *r*_1_ and *r*_2_ (per protein molecule concentration, mM^−1^s^−1^) for HSA-NIT conjugates per magnetic field. (**B**) *T*_1_- and *T*_2_-weighted MRI images of HSA-NIT containing phantoms at 25 °C in a 3 T MRI scanner in three HSA-NIT (Reproduced from Int. J. Mol. Sci. 2024, 25, 4041 [120]); open access article distributed under the terms and conditions of the Creative Commons Attribution (CC BY 4.0).

The lowest reduction rate constant (k = 0.0007 M^−1^s^−1^) was obtained for HSA-Et/Et-mal. Consequently, albumin-based conjugates incorporating sterically hindered tetraethyl-substituted nitroxide derivatives demonstrate substantially enhanced stability, enabling prolonged maintenance of paramagnetic properties under physiological reducing conditions. The relaxivities *r*_1_ and *r*_2_ of HSA-NIT conjugates were evaluated at 1.88 T, 3 T, 7 T, and 14.1 T to provide data compatible with all types of known medical MRI spectrometers (Figure 8A). The phantoms at 3 T are presented in Figure 8B (for 14.1 T phantoms, see the paper [120]). The *r*_1_ value increased from 1.88 T to 3 T and then decreased. On the contrary, *r*_2_ increased and, at 14.1 T, decreased dramatically. The explanation of this phenomenon requires additional experiments on high-field MRI scanners. The *r*_2_*/r*_1_ values increased with the field, indicating r_2_ behavior at a high field. Among the conjugates, HAS-Et/Et-mal demonstrated the highest relaxivities both per molecule and per nitroxide residue, with tetraethyl-substituted nitroxides outperforming tetramethyl variants. Phantom MRI studies at 3 T (Figure 8B) confirmed effective signal registration at clinically relevant protein concentrations (0.005–0.5 mM), producing contrast comparable to that of dendrimeric and BASP-based ORCAs and enabling dual-mode *T*_1_*/T*_2_-weighted imaging. Combined with excellent solubility, stability, biocompatibility, and albumin’s intrinsic tumor-targeting capability via the Enhanced Permeability and Retention effect, these HAS-NIT conjugates represent a promising platform for metal-free cancer MRI. Biomacromolecular ORCAs leverage the inherent advantages of biological molecules to overcome key limitations of low-molecular-weight nitroxides. By combining high spin density, resistance to bioreduction, and tumor-selective accumulation via passive or active transport, these platforms achieve molecular relaxivities that rival or exceed those of clinical Gd-based CAs, making them an attractive metal-free alternative for sensitive and safe cancer MRI.

### 2.4. Comparison of Different Types of Nitroxides and Studies’ Limitations

Different types of nitroxides presented above represent a promising alternative to conventional metal-based CAs. However, most of them have their own advantages and limitations. The important parameters, such as relaxivity, tumor accumulation selectivity, blood circulation time, and biocompatibility behavior, are addressed in Table 8. Based on all factors, Brush-Arm Star Polymers and biomacromolecules are the most promising for further use and translation into clinics (Table 8).

The essential factor for these platforms that must be discussed is selective accumulation in the tumor tissue. The ORCA can be excellent in terms of physical properties, but if it is not delivered to the tumor, it is useless. Three main types of accumulation are possible: via (1) passive targeting and the Enhanced Permeability and Retention (EPR) effect; (2) active targeting; and (3) physical/stimuli-responsive targeting. In an ideal situation, all of them should be used together. The EPR effect represents a pathophysiological phenomenon that underpins the passive targeting of large molecules and nanoparticles to solid tumors [130]. The mechanism relies on two complementary features of tumor pathophysiology: (1) abnormal tumor vasculature characterized by hyperpermeable vessels and enlarged interendothelial gaps that facilitate the extravasation of macromolecules [131] and (2) impaired lymphatic drainage within tumor tissue, which prevents efficient clearance of extravasated agents and results in their prolonged retention [132]. Multiple clinical studies confirm that macromolecular accumulation occurs in human tumors, albeit with varying intensity depending on tumor type, anatomical location, and the state of the tumor microenvironment [132]. For active targeting, targeting moieties, such as reporter groups, ligands, antibodies, or peptides, should be attached to the carrier to bind specifically to receptors overexpressed on cancer cell surfaces. One general example of these groups is folate and sugar residues. A physical targeting example is the transport of MNPs into cancer tissue using external magnetic fields. Stimuli-responsive molecules release a drug or probe at the tumor using different triggers, such as the tumor’s acidic pH, high temperature, or specific enzymes, as presented in the next section for OMRI agents. Most polymer-based ORCAs presented above lack active transport residues, which should be improved in future work in the area. The mechanism of their transport is the EPR effect, driven by their large size. On the contrary, most biomolecule-based ORCAs are transported in passive and active pathways due to interactions with various receptors. For example, HSA-NIT conjugates, based on plasma albumin protein, can be delivered due to the high MW of albumin and its long circulation time, and also by interaction with the neonatal Fc receptor (FcRn), SPARC, and gp18, gp30, and gp60 [120,129].

Another limitation is the lack of comprehensive relaxivity studies of ORCAs. In most papers focused on the development and in vivo application of new CAs, relaxivity values are measured at magnetic field strengths (B_0_) from 1.5 to 3.0 T, which are used in most clinical MRI scanners [133,134]. In rare cases, 7 T and 17 T scanners are used for brain MRI. However, as shown in Table 1, Table 2, Table 3, Table 4, Table 5, Table 6 and Table 7, very different magnetic field strengths (0.2–14.1 T) were used in relaxivity studies. Most research groups studied relaxivities only at one magnetic field, 3 T [79,82,87]. This happened because most labs have only one device with a single magnetic field. In most cases, it is a Bruker NMR spectrometer with a field of ~7 T. That is why, for negligible ORCA, the relaxivity is known in the “medical” range of 0.5–3 T. This difference in measurement conditions can lead to significant changes in the interpretation of CA efficiency and difficulties in comparison. An extensive investigation of relaxivities as a function of field from 0.5 T to at least 3 T (better 14 T) is required in the area to understand the behavior and physical mechanism of ORCA’s relaxivity. High field strengths can be promising for polymer-based ORCAs and *T*_2_-weighted MRI. According to Table 7 and Figure 8, *r*_2_ values are significantly higher, enabling good-quality contrast. The optimized polymer-based ORCA’s design and its field-dependent relaxivity allow the use of the same conjugate in one field range for *T*_1_ contrast, in another for both *T*_1_ and *T*_2_, and at ultra-high (≥7.0 T) magnetic fields for *T*_2_ images.

## 3. Application of Nitroxides for OMRI Techniques

Standard ^1^H-MRI suffers from low sensitivity, limiting its ability to detect small tumors and perform in vivo redox or metabolic imaging [135]. Signal enhancement in ^1^H-MRI can be significantly improved by combining CAs with physical hyperpolarization techniques [136]. Among the most promising approaches for clinical diagnostics using free radicals are Overhauser dynamic nuclear polarization (ODNP) [124,125] and ODNP-enhanced MRI (OMRI) [137,138]. For tumor imaging via OMRI, specialized hyperpolarizing CAs are required. These agents include nitroxides [118,138,139], which facilitate efficient polarization transfer, resulting in ^1^H signal enhancements that usually do not exceed 100-fold (Table 9). The method requires transferring spin order from free electrons to nearby protons in water, which requires an RF field at a frequency near the electron Larmor frequency. The high-power RF leads to overheating, which is unsafe for in vivo experiments. In this way, a low field range is preferable. The key parameters characterizing the performance of CAs in OMRI are the maximum enhancement factor (|E_max_|) and the radiofrequency power required to achieve half of the maximum enhancement (P_1/2_). |E_max_| represents the theoretical upper limit of proton MR-signal enhancement achievable through polarization transfer from the unpaired electrons of the ORCAs to surrounding water nuclei. A higher |E_max_| directly translates into greater sensitivity and image contrast in OMRI. P_1/2_ characterizes the efficiency of electron spin saturation: a lower P_1/2_ indicates that the EPR transition can be effectively saturated at low radiofrequency power levels. It is critically important for in vivo applications, as it minimizes the specific absorption rate and tissue heating. The optimal OMRI ORCAs combine high |E_max_| with low P_1/2_ [120,139], thereby enabling strong signal enhancement under safe, low-power irradiation conditions suitable for biological systems.

The unique redox properties of nitroxides make them promising for imaging oxidative stress [138,140,142]. In mice bearing C6 gliomas, intravenous injection of 3-CP resulted in a 2.7-fold increase in tumor tissue and an 8.4-fold increase in the OMRI signal in major blood vessels at 0.2 T [143]. The temperature elevation is not to exceed 1 °C during the procedure. However, low-molecular-weight nitroxides exhibit several limitations as ORCAs, as discussed in detail in Section 2.1. In the context of OMRI, its primary disadvantages include rapid bioreduction in tissues, rapid renal clearance, and a lack of selectivity for tumor tissue [51,60]. As an alternative, sterically shielded nitroxides, such as tetraethyl-substituted derivatives, can be employed due to their enhanced resistance to bioreduction. Sterically shielded 2,2,5,5-tetraethyl-PROXYL has demonstrated approximately a 1.5-fold increase in P_1/2_ and a significant reduction in the maximum enhancement factor |E_max_| to 62.6 [139]. Specifically, ^15^N labeling enhances signal intensity, resulting in a 17% increase in proton polarization compared to their unlabeled counterparts [144]. Moreover, dual isotopic labeling, the simultaneous incorporation of ^15^N and ^2^H, further amplifies the signal, yielding an approximately 70% increase in the enhancement factor. Notably, these doubly labeled nitroxides exhibit the lowest P_1/2_ value of 1.1 W, 5–6-fold lower than that of unlabeled analogs, achieving a maximum |E_max_| of 126.2 [139]. These findings highlight the promise of ^15^N–labeled nitroxides for highly sensitive imaging, particularly when combined with deuteration. To address the limitations of low selectivity and rapid bioreduction inherent to low-molecular-weight nitroxides, they can be incorporated into various polymeric nanostructures to develop ORCAs for OMRI. Dendritic poly(L-lysine)-based ORCAs were evaluated for OMRI applications [118]. The in vivo OMRI enhancement factor for the dendrimer-conjugated agent was found to be 4.9, whereas the 3-carboxy-PROXYL yielded a higher enhancement factor of 8.6. This reduction in the enhancement factor arises because the covalent attachment of nitroxides restricts solvent accessibility and molecular mobility, increases the rotational correlation time, broadens its EPR spectrum, and negatively impacts the Overhauser enhancement efficiency. One of the main strategies for improving ORCA performance in OMRI is optimizing the mobility of spin probes and their accessibility to water molecules. In this context, the biomacromolecule heparin, owing to its high hydrophilicity and chain flexibility, acts as an effective polymeric scaffold [141]. Heparin–nitroxide conjugates use these properties to achieve significant signal enhancement, with an |E_max_| reaching 27.0.

**Figure 9:**
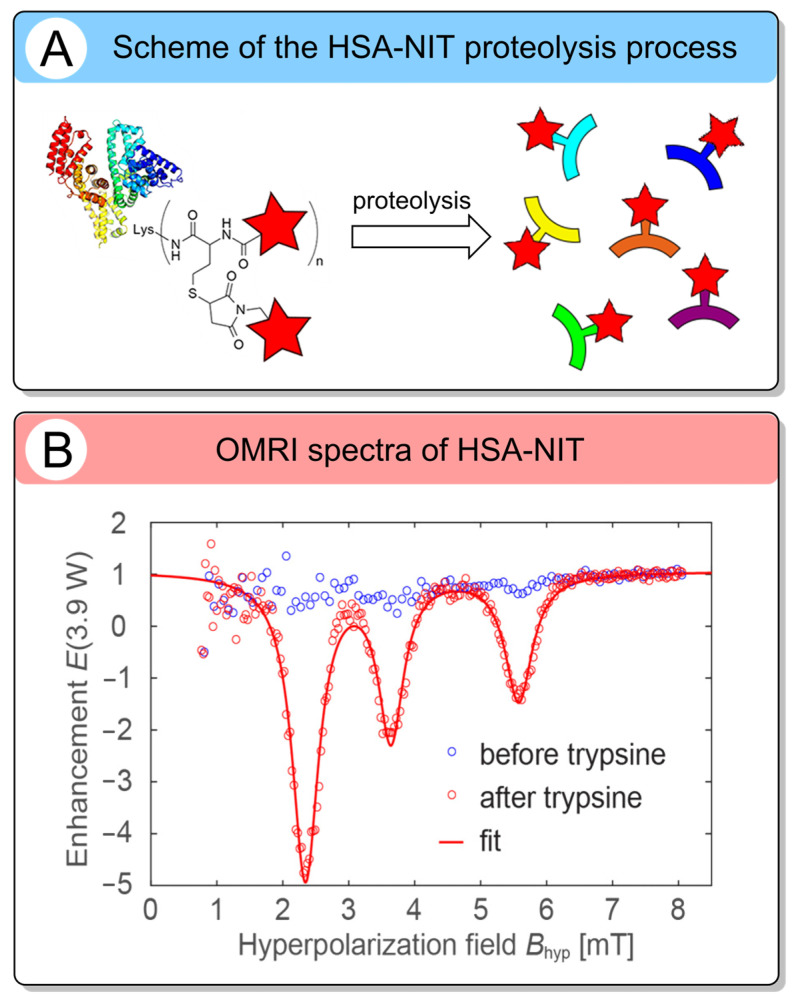
(**A**) Schematic process of the HSA-NIT proteolysis. (**B**) OMRI spectra of HSA-NIT before and after the cleavage process (~20 h after injection of trypsin). The red star represents a nitroxide residue (reproduced from Int. J. Mol. Sci. 2024, 25, 4041 [120]); open access article distributed under the terms and conditions of the Creative Commons Attribution (CC BY 4.0).

An even more promising approach involves stimuli-responsive delivery systems that release low-molecular-weight nitroxides specifically within the tumor microenvironment through enzymatic cleavage [64,135,145]. A representative example is HSA-NIT [120], an albumin–nitroxide conjugate, discussed in Section 2.3, which is designed to be degraded by proteases overexpressed in tumors (Figure 9). Upon proteolytic cleavage, HSA-NIT yields small, highly mobile nitroxide fragments, resulting in an |E_max_| of 40–55 at low saturation power P_1/2_ 10–12 W. This performance exceeds that of previously reported polymeric ORCAs. However, it remains nearly 2-fold lower than that of free low-molecular-weight nitroxides in terms of |E_max_|. Nevertheless, because albumin selectively accumulates in tumors via the EPR effect and receptor-mediated uptake, HSA-NIT can be a promising in vivo CA.

## 4. Future Perspectives and Conclusions

Nitroxides represent a promising alternative to conventional metal-based CAs for MRI-based cancer diagnostics due to their low toxicity, excellent biocompatibility, and broad chemical functionalization options. These properties make ORCAs particularly attractive for developing safer next-generation MRI contrast agents. Despite these advances, several fundamental challenges still limit the clinical translation of ORCAs. A key limitation is the significantly low relaxivity of nitroxides compared to gadolinium-based chelates [51,52]. Moreover, nitroxides are susceptible to rapid reduction, leading to the formation of diamagnetic hydroxylamines under physiological conditions [47,48,49,50]. Lack of selective accumulation in tumor tissues, rapid elimination from the body, and incomplete understanding of pharmacokinetics and metabolism further complicate their application [82]. To address these challenges, significant progress has been made in the development of ORCAs in recent years through rational molecular design and the conjugation of nitroxides to macromolecular platforms. Replacing methyl groups at the α-position relative to the nitroxyl radical with bulkier ethyl or spirocyclohexyl substituents has reduced the rate of bioreduction by up to 60-fold compared to conventional analogs such as TEMPO or PROXYL [74,75,76,77,78]. Furthermore, conjugation of nitroxides to polymeric carriers, including dendrimers, linear polymers, brush-arm star polymers, and biomacromolecules, simultaneously solves several main challenges: (1) slowing molecular tumbling of the nitroxide due to increased steric hindrance enhances both longitudinal and transverse relaxivity by 2–3-fold per nitroxyl radical [96]; (2) increasing the local concentration of nitroxides in the polymer, as exemplified by BASP-ORCA3, yields exceptional relaxivities of *r*_1_ > 100 mM^−1^s^−1^ and *r*_2_ > 1000 mM^−1^s^−1^ per molecule [41]; (3) the bulky structure of the carrier provides steric shielding that restricts access of endogenous reductants, thereby substantially improving nitroxide stability in vivo. Moreover, incorporation of functional groups such as polyethylene glycol (PEG) and folic acid into polymeric ORCAs enhances aqueous solubility, prolongs systemic circulation, and promotes tumor accumulation through both passive and active targeting mechanisms. Preclinical studies have confirmed that modern nitroxide-based systems can rival Gd-based CAs in contrast enhancement for tumor imaging [41,95,119]. However, only a few systems are known to effectively target tumor tissue, due to the field’s current focus on improving the physical parameters of the structure itself. It will take some time to further improve and refine the design to achieve the desired effect. Nevertheless, translating these laboratory developments into clinical practice requires addressing several key issues: standardizing synthesis, conducting comprehensive pharmacokinetic investigations, assessing long-term biocompatibility and metabolic pathways, and developing carriers with tumor-type–specific targeting capabilities.

Beyond conventional MRI applications, ORCAs show considerable promise for multimodal imaging and theranostic (therapy + diagnostics) construction production. Polymeric ORCAs can be easily combined with fluorescence imaging or loaded with drugs, enabling multifunctional platforms for personalized medicine. Another important direction involves advanced imaging strategies. Currently, methods to improve the accuracy of cancer diagnosis are being actively developed using physical hyperpolarization techniques. Of particular significance are strategies that employ nitroxides as hyperpolarizing agents in Overhauser-enhanced MRI [123,124], thereby enhancing image contrast by approximately 100-fold and providing functional information on tumor status [138,139]. Moreover, integrating nitroxides into stimulus-responsive biomacromolecules, such as HSA-NIT, or using other cleavable bonds, enables the development of probes activated by enzymes overexpressed in the tumor microenvironment, thereby significantly improving both the specificity and the diagnostic value of the OMRI technique [120]. Stimulus-responsive systems that activate under specific biological conditions, such as low pH in cancer tissue or lysosomes, high enzyme activity, or redox changes, are being actively developed to improve imaging specificity and diagnostic accuracy. Taken together, ORCAs have significant potential for further development by integrating advanced MRI methodologies with rationally engineered polymeric platforms that deliver high local concentrations of nitroxides and tumor-targeting ligands. Although ORCAs have not yet reached the stage where they can replace traditional metal-based CAs, continuous advances in design, polymer chemistry, and imaging methodologies are steadily overcoming their current limitations. However, to translate these metal-free CAs into routine clinical practice, comprehensive preclinical and clinical studies are essential to validate their safety, efficacy, pharmacokinetics, biodegradation pathways, long-term biocompatibility, and diagnostic performance in patients. With further interdisciplinary efforts, ORCAs may lead to a new generation of safer platforms for further production of multifunctional imaging or theranostics agents for clinics.

## Figures and Tables

**Table 1:** Relaxivities *r*_1_ and *r*_2_, and reduction constant (*k*_red_) of common low-molecular-weight nitroxides, which were studied for MRI application.

Nitroxide	Magnetic Field Strength, T	*r*_1/_*r*_2_, mM^−1^s^−1^	*k*_red_, M^−1^s^−1^	^1^H-MRI Application	Ref.
TEMPO	1.4	0.15/0.10	-	in vitro MRI phantoms	[62]
TEMPOL	1.0	0.26/-	5.6 ± 0.2	in vitro MRI phantoms	[63]
	7.0	0.14/-			
Oxo-TEMPO	0.2	0.50/-	-	in vitro MRI phantoms	[64]
3-carboxy-PROXYL	7.0	0.20/0.23	0.063 ± 0.002	-	[65,66]
		0.18/0.20			
2,2,5,5-tetraethyl-PROXYL	1.4	0.21/0/25	<0.001	-	[67]
SpiroHex	7.0	0.21/0.30	0.031 ± 0.003	in vitro MRI phantoms	[38]
MCP	1.0	0.27/-	-	-	[51,52]
	4.7	0.16/-			
	7.0	0.14/-			
CMP	4.7	0.17/-	-	in vivo MRI of mice bearing squamous carcinoma	[51]
Gd-DTPA * Magnevist	1.5	3.8/5.2	-	-	[68,69]
	3.0	3.3/5.0			
	7.0	3.1/-			
Gd-DOTA * Dotarem	1.5	3.9/-	-	-	[70]
	3.0	3.4/-			
	7.0	2.8/-			

* *T*_1_-weighted Gd-based contrast agents for comparison.

**Table 3:** Functionalized nitroxide structures, which can form nanosystems in water solution, their relaxivities *r*_1_ and *r*_2_, size, and some MRI applications.

Nitroxide, Size of Nanosystem (nm) *	Magnetic Field Strength, T	*r*_1_*/r*_2_, mM^−1^s^−1^ per Molecule	*r*_1_*/r*_2_, mM^−1^s^−1^ per Nitroxide	^1^H-MRI Application	Ref.
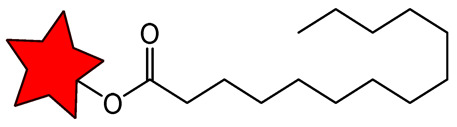 Myristic acid nitroxide phytantriol, 226 nm	3.0	0.59/1.35	-	in vivo MRI of Sprague Dawley rat	[93]
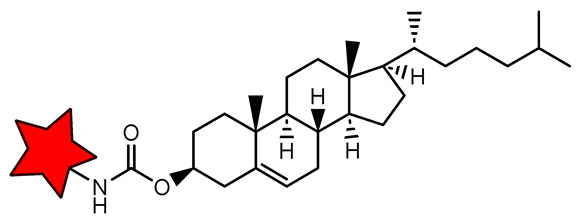 Cholesterol nitroxide phytantriol, 224 nm	3.0	1.08/2.21	-	in vivo MRI of Sprague Dawley rat	[39]
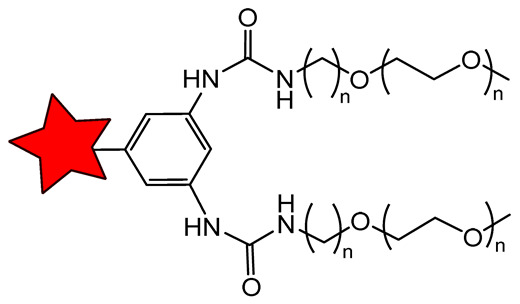 TEMDO-UBD (RNP 2), 200 nm	1.0	-	0.29/0.26	in vivo MRI of BALB/c mice bearing colon-26 tumors	[89]
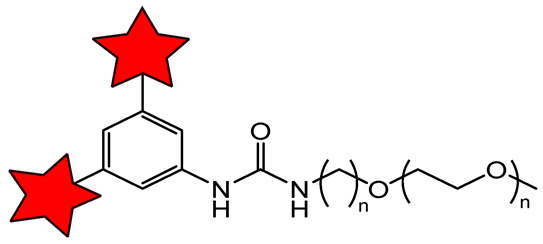 TEMDO-UBD (2-HEG), 450 nm	1.0	-	0.41/0.42	in vitro MRI phantoms	[90]

* The red star is a schematic representation of a nitroxide residue.

**Table 4:** Linear polymer-based ORCAs, their relaxivities *r*_1_ and *r*_2_, size, and some MRI applications.

ORCA, Size (nm) *	Magnetic Field Strength, T	*r*_1_/*r*_2_, mM^−1^s^−1^ per Nitroxide	*r*_1_/*r*_2_, mM^−1^s^−1^ per Molecule	MRI Application	Ref.
Polyurethane-TEMPO, 70 nm	1.5	0.66/0.98	~10/15	in vitro MRI phantoms	[101]
PEG-b-polycarbon- ate-based copolymers-PROXYL, 103 nm	3.0	0.22/-	2.4/-	in vivo MRI of C57BL/6 J mice	[96]
Glucose-PEO-poly-(L-lactic acid)-TEMPO, 281 nm	3.0	0.3/-	0.3/-	in vivo MRI of mice bearing tumor cells Hep1-6	[102]
Hydrogel with TEMPO-PEG, poly(acrylic acid), and agarose	7.0	0.30/-	-	in vivo MRI of mice	[105]

* Hydrodynamic diameter of ORCAs measured by dynamic light scattering.

**Table 5:** BASP ORCAs, relaxivities r_1_ and r_2_, size, and MRI applications.

ORCA, Size (nm) *	Magnetic Field Strength, T	*r*_1_/*r*_2_, mM^−1^s^−1^ per Nitroxide	*r*_1_/*r*_2_, mM^−1^s^−1^ per Molecule	^1^H-MRI Application	Ref.
Polyacetylene-TEMPO	3.0	0.27/-	3.3/-	in vivo MRI BALB/c mice bearing HeLa tumor cells	[107]
pDHPMAmPEG-PpaPROXYL (branched), 28 nm	3.0	0.50/-	4.7/-	in vivo MRI of BALB/c mice bearing 4T1 tumor cells	[96]
pDHPMAmPEGPpaPROXYL (linear), 23 nm	3.0	0.93/-	10.5/-		[102]
PCE-mPEG-Ppa-PROXYL, 12 nm	3.0	0.64/-	1.2/-	in vivo MRI of BALB/c mice bearing	[108]
ORCAFluors, 19 nm	7.0	0.32/0.82	37/95	in vivo MRI of BALB/c mice	[106]
BASP-ORCA1, 31 nm	7.0	0.41/4.67	37.6/428.8	in vivo MRI of NCr/NU mice	[38]
BASP-ORCA3, 29 nm	7.0	0.63/4.62	~126/>1000	in vivo MRI of SCID mice bearing myeloma	[41]
Pro ORCA BASPs, 25 nm	7.0	0.26/4.85	~20/380	in vivo MRI NCr/NU mice bearing A549 tumor	[98]
Fe_3_O_4_/Feraheme **	1.5	-	20/61	-	[38,111,112]
	3.0		10/88		
	7.0		-/68		
Fe_3_O_4/_Resovist **	1.5	-	25.4/151	-	[113,114]
	3.0		9.7/189		

* Hydrodynamic diameter of ORCAs measured by dynamic light scattering. ** *T*_2_-weighted Gd-based contrast agents for comparison.

**Table 6:** Dendrimeric polymer-based ORCAs with generation number, their relaxivities *r*_1_ and *r*_2_, and MRI applications.

ORCA	Dendrimer Generation	Magnetic Field Strength, T	*r*_1_/*r*_2_, mM^−1^s^−1^ per Nitroxide	*r*_1_/*r*_2_, mM^−1^s^−1^ per Molecule	^1^H-MRI Application	Ref.
Polyamidoamine (PAMAM)	0–1	0.5	0.11–0.13/-	1.3–1.6/-	-	[116,118]
Polypropylenimine (PPI)	2–4	7.0	0.29–0.42/-	3.8–13.1/-	in vivo MRI C57BL/6 mice	[44]
Poly (L-lysine)	6	1.5	0.50–0.80/-	4–10/-	in vivo MRI of ddY mice bearing 4T1 cells	[117]
Oligoethylene glycol (OEG)	0–1	7.0	0.17–0.18/ 0.19–0.20	0.9–3.4/ 1.0–4.0	-	[66]
Polyphosphorhydrazone (PPH)	0–3	7.0	0.23–0.27/ 0.24–0.33	1.4–12.9/ 1.4–16.0	in vivo MRI of GL261 GB-bearing mice	[65,95]

**Table 7:** Biomacromolecule-based ORCAs, their relaxivities *r*_1_ and *r*_2_, and some MRI applications.

ORCA	Magnetic Field Strength, T	*r*_1_/*r*_2_, mM^−1^s^−1^ per Nitroxide	*r*_1_/*r*_2_, mM^−1^s^−1^ per Molecule	^1^H-MRI Application	Ref.
CS-TEMPO-FA	0.5	2.63/3.47	-	in vivo MRI of mice bearing 4T1 cells	[46,119]
	1.5	1.58/2.11	254/340		
HSA-Me	1.9	0.30/0.9	3.0/9	in vitro MRI phantoms	[120]
	3.0	0.38/1.2	3.5/11		
	7.0	0.20/1.4	2.7/18		
	14.1	0.12/1.9	1.6/26		
HSA-Et	1.9	0.39/1.7	4.0/18	in vitro MRI phantoms	[120]
	3.0	0.50/2.1	4.8/22		
	7.0	0.23/2.2	2.9/29		
	14.1	0.14/2.9	1.8/38		
HSA-Me/Me-mal	1.9	0.33/0.9	6.3/17	in vitro MRI phantoms	[120]
	3.0	0.48/1.3	8.2/22		
	7.0	0.30/2.0	5.2/34		
	14.1	0.24/2.2	4.1/38		
HSA-Et/Et-mal	1.9	0.42/1.2	11.0/32	in vitro MRI phantoms	[120]
	3.0	0.38/1.3	15.1/50		
	7.0	0.43/4.2	10.0/97		
	14.1	0.29/3.1	6.7/73		
TMV-TEMPO	1.5	1.5/4.7	~270/840	in vitro MRI phantoms	[121,122]
	9.4	0.4/5.2			

**Table 8:** Advantages and disadvantages of ORCAs.

ORCA Type and Number of Nitroxides	Relaxivity *	Tumor Accumulation Selectivity **		Circulation Time	Biocompatibility **	Advantages (+)/Limitations (−)
	r_1_/r_2_	ERP-Effect	Targeting			
Low-MW 1–10	−/−	−	− or +	Short	+	+ Cheap and easy synthesis − Rapid reduction and elimination; short contrast period
Linear Polymer 5–20	+/+	− or +	− or +	Moderate	+ with PEGylation	+ Moderate or high relaxivity and radical stability − Hard and expensive polymer synthesis; poor aqueous solubility due to the high modification by nitroxides
Brush-Arm Star Polymer 10–200	++/+++	+	− or +	Moderate	+ with PEGylation	
Dendrimeric Polymer 16–64	+/+	− or +	− or +	Moderate	+	
Biomacromolecule 10–200	++/+++	++	++	Long	++	+ Moderate or high relaxivity, good or excellent nitroxide stability, biodegradability, and low immunogenicity − Complex synthesis with preservation of biological properties

* Low − (~0.1–2 mM^−1^s^−1^), medium + (2–20 mM^−1^s^−1^), high ++ (20–200 mM^−1^s^−1^), extremely high +++ (300–1000 mM^−1^s^−1^). ** No −, medium +, high ++.

**Table 9:** Nitroxides for OMRI, maximum enhancement factor |E_max_|, radiofrequency power required to achieve half of the maximum enhancement P_1/2_, and MRI applications.

ORCA	P_1/2_, W	|E_max_|	OMRI Application	Ref.
TEMPO	6.2	153.7	in vitro OMRI	[139]
	6.1	101.9		
TEMPOL	7.1	91.1	in vivo OMRI of Sprague-Dawley rat	[138,139]
PROXYL	4.7	107.9	in vitro OMRI	[139]
3-carbamoyl-PROXYL (3-CP)	-	101.0	in vivo OMRI of mice bearing RKO colorectal cancer	[140,141]
	5.3	88.1		
2,2,5,5-tetraethyl-PROXYL	7.8	62.6	in vitro OMRI	[140]
SL-heparin	-	27.0	in vitro OMRI	[142]
HSA-NIT	10.8	54.0	in vitro OMRI	[120]

## Data Availability

No new data were created or analyzed in this study.

## Abbreviations

The following abbreviations are used in this manuscript:

BASP	Brush-arm star polymer
CD	β-cyclodextrin
EPR	Electron paramagnetic resonance
EPR effect	Enhanced Permeability and Retention effect
HSA	Human serum albumin
MNP	Iron oxide nanoparticles
MRI	Magnetic resonance imaging
ODNP	Overhauser dynamic nuclear polarization
OMRI	Overhauser-enhanced magnetic resonance imaging
ORCA	Organic radical contrast agent
PROXYL	2,2,5,5-tetramethylpyrrolidin-1-oxyl
TEMPO	2,2,6,6-tetramethylpiperidin-1-oxyl
TEEPO	2,2,6,6-tetraethylpiperidin-1-oxyl
